# Estimation of heritabilities and additive genetic correlations for reproduction traits in swine: insights for tropical commercial production systems using multiple trait animal models

**DOI:** 10.5713/ab.23.0163

**Published:** 2023-08-23

**Authors:** Udomsak Noppibool, Thanathip Suwanasopee, Mauricio A. Elzo, Skorn Koonawootrittriron

**Affiliations:** 1 Department of Animal Science, Faculty of Agriculture and Technology, Rajamangala University of Technology Isan Surin Campus, Surin 32000, Thailand; 2 Department of Animal Science, Faculty of Agriculture, Kasetsart University, Bangkok 10900, Thailand; 3 Department of Animal Sciences, University of Florida, Gainesville, FL 32611-0910, USA

**Keywords:** Genetic Parameters, Multiple Trait Model, Reproduction Traits, Swine

## Abstract

**Objective:**

This study was to estimate heritabilities, additive genetic correlations, and phenotypic correlations between number of piglets born alive (NBA), litter birth weight (LTBW), number of piglets weaned (NPW) and litter weaning weight (LTWW) in different parities of Landrace (L), Yorkshire (Y), Landrace×Yorkshire (LY), and Yorkshire×Landrace (YL) sows in a commercial swine operation in Northern Thailand.

**Methods:**

Two models were utilized, a single trait repeatability model (RM) and a multiple trait animal model (MTM). The RM assumed reproductive records from different parities to be repeated values of the same trait, whereas the MTM assumed these records to be different traits. The two models accounted for the fixed effects of farrowing year-season, genetic group of the sow, heterosis, and age at first farrowing, and the random effects of sow, boar, and residual.

**Results:**

Heritability estimates from RM were 0.02±0.01 for NBA, 0.10±0.01 for LTBW, 0.04±0.01 for NPW, and 0.11±0.01 for LTWW. Heritability estimates from MTM fluctuated across parities, ranging from 0.04±0.01 in parity 2 to 0.09±0.02 in parity 4 for NBA, 0.07± 0.02 in parity 2 to 0.16±0.02 in parity 3 for LTBW, 0.04±0.02 in parity 4 to 0.08±0.01 in parity 1 for NPW, and 0.16±0.02 in parity 1 to 0.20±0.02 in parity 2 for LTWW. Additive genetic correlation estimates from MTM were also variable, ranging from 0.29±0.24 between NBA in parity 1 and NBA in parity 2 to 0.99±0.05 between LTWW in parity 3 and LTWW in parity 4.

**Conclusion:**

The findings of this study highlight the advantage of using MTM for the genetic improvement of reproductive traits in swine and contribute to the development of sustainable swine breeding programs in Thailand.

## INTRODUCTION

Reproduction traits are critical factors for maximizing sow productivity and ensuring the sustainability of commercial swine operations. Key traits such as the number of piglets born alive (NBA), litter birth weight (LTBW), number of pigs weaned (NPW), and litter weaning weight (LTWW) play pivotal roles in swine selection programs across diverse temperate and tropical environmental conditions [1–3]. In Thailand, genetic evaluation for reproduction traits relies on the widely employed best linear unbiased prediction (BLUP) methodology. However, continuous efforts are essential to enhance the efficiency of swine genetic evaluation by carefully considering alternative approaches for estimating genetic parameters. This necessitates ensuring the effectiveness of genetic estimation procedures and gaining a comprehensive understanding of the genetic associations between traits, which are crucial for the successful implementation of genetic improvement programs.

Repeatability models have conventionally served as a cornerstone in swine genetic evaluations, capitalizing on the availability of repeated records of productive performance and pedigree data [4–7]. These models assume full additive genetic correlations between reproduction records in the first parity and subsequent parities [4,8]. Consequently, when reproduction traits in the first parity exhibit strong correlations with those in later parities, it is assumed that the records across all parities represent multiple observations of a single trait. However, in cases where repeatability is low, indicating variation among animal developmental stages, reproduction records in the first and subsequent parities are treated as distinct traits. This scenario commonly calls for the adoption of multiple trait animal models (MTM) [7,9]. By treating each parity as a separate trait, MTM provide a more accurate and comprehensive approach to the genetic evaluation of reproduction traits in swine. These models account for the inherent incomplete additive genetic correlations between records from different parities, thereby offering a more refined understanding of the underlying genetic architecture.

Previous studies [5,7,9–12] have demonstrated the utility of MTMs for estimating genetic parameters for reproduction traits. By incorporating the complexities associated with different parities, these models provide valuable insights into the genetic control of reproduction traits at various stages of sow development. To develop an effective swine genetic breeding program in Thailand, it is paramount to compare the genetic parameters obtained through the application of both repeatability models and MTMs within Thai swine populations. This comparative analysis is instrumental for assessing the advantages and potential improvements offered by the utilization of MTMs.

Therefore, the primary objective of this research was to conduct a comprehensive comparison of genetic parameters for NBA, LTBW, NPW, and LTWW across different parities within a commercial swine population in Northern Thailand. By employing both the traditional repeatability model and the more sophisticated MTMs, this study aims to elucidate the benefits and suitability of MTM in the context of swine genetic evaluation under Thai environmental conditions. Emphasizing the advantages of the MTM approach, this research seeks to provide compelling evidence of its effectiveness in capturing the complexity and variability associated with swine reproduction traits in Thailand. The outcomes of this study hold significant implications for advancing swine genetic evaluation practices because they highlight the potential advantages offered by MTMs. By demonstrating their suitability and enhanced accuracy in capturing the underlying genetic architecture, this research contributes to the ongoing efforts aimed at refining genetic selection strategies for reproduction traits. Ultimately, these advancements have the potential to drive more precise and reliable breeding programs, leading to improved sow productivity and the establishment of sustainable and efficient swine production systems.

## MATERIALS AND METHODS

The sows utilized in this study were sourced from the reproductive herd of a commercial pig farm, which adhered to the good agricultural practices as prescribed by the National Bureau of Agricultural Commodity and Food Standards. The performance data of the sows were extracted from a comprehensive and regularly updated database maintained by the farm. Ethical clearance for the study was obtained from the Institutional Animal Care and Use Committee of Kasetsart University, with approval number ACKU60-AGR-007.

### Data, animals, and traits

This study was approved by the IACUC of Kasetsart University (approval number: ACKU60-AGR-007). The data for this study were obtained from a swine database recording system (PigCHAMP) in a single farm located in Northern Thailand. The dataset comprised sow reproduction records from the first to the last or tenth parity containing identification number, breed group, sire, dam, birth date, farrowing date, weaning date, NBA, LTBW, NPW, and LTWW. To ensure data quality, the original dataset of 14,604 sows underwent thorough editing to remove records with missing, erroneous, or incomplete information. Sows without recorded birth date, farrowing date, or weaning date were excluded, as well as gilts with a first farrowing before 350 days or after 550 days. Only litters with at least first parity records were included in the analysis, and parities greater than 10 were excluded. Approximately 33% of records were excluded from the original dataset due to incomplete information. This rigorous data editing process ensured the reliability and integrity of the dataset and set the foundation for robust and meaningful analyses. The final edited dataset encompassed 49,145 reproduction records from 9,830 sows and 1,359 boars. Reproduction data were gathered from July 1989 to December 2013. Four breed group of sows were included in the analysis: Landrace (L; n = 2,124), Yorkshire (Y; n = 724), Landrace×Yorkshire (LY; n = 2,650), and Yorkshire×Landrace (YL; n = 4,332). Purebred L and Y sows were the progeny of 640 sires (395 L and 245 Y) and 1,319 dams (895 L and 424 Y), and crossbred LY and YL sows were the progeny of 969 sires (608 L and 361 Y) and 2,674 dams (1,608 L and 1,066 Y). A total of 260 Duroc (D) boars were represented in the dataset for the three-breed terminal crosses. Four reproduction traits (NBA, LTBW, NPW, and LTWW) were considered for analysis.

### Climate, nutrition, and management

This study was conducted at a single commercial swine farm located in Northern Thailand, between latitude 18° 47′ 43″ North and longitude 98° 59′ 55rime; East, at an elevation of 310 meters above sea level. The farm experienced three distinct seasons, namely winter (November to February), summer (March to June), and rainy (July to October). Over the course of the 24-year study period, the average outdoor temperature in this region ranged from an average minimum of 17°C to an average maximum of 35°C, with relative humidity ranging from an average minimum of 37% to an average maximum of 99%. The average annual rainfall varied from an average minimum of 880 mm to an average maximum of 1,457 mm over the past thirteen years.

Gilts and sows were reared in an open-house system equipped with water drippers, sprinklers, and fans, while boars were housed in an evaporative cooling system (EVAP) to mitigate the effects of the tropical climate. Females that had their first farrowing in the same year-season were assumed to have received similar feeding and management. *Ad-libitum* water was provided to the animals through water nipples. Gilts and non-lactating sows were fed twice a day and had an approximate intake of 2.5 kg/d of feed containing 16% crude protein and 3,200 to 3,500 kcal/kg. Nursing sows were fed four times a day and had an approximate intake of 5 to 6 kg/d of feed containing 17% to 18% crude protein and 4,060 kcal/kg.

Estrus was detected twice daily utilizing the back-pressure test and boar exposure as standard protocols. Gilts and sows exhibiting signs of standing heat in front of the boar, along with clear reddening and swelling of the vulva, were identified as being in estrus. Artificial insemination was the sole method of mating employed in this study. Gilts were mated after their third observed estrus or at 8 to 9 months of age, or when their body weight reached approximately 140 kg. Sows were mated after their second observed estrus. The first insemination was carried out within 12 hours of the onset of estrus, followed by a second insemination 12 hours later. Sows were housed in individual stalls during mating and gestation and kept in individual pens along with their litters during lactation. Pregnant gilts and sows were housed in gestating stalls until 7 days prior to being moved to farrowing pens. Sows were transferred to mating stalls after weaning. Piglets were weaned when they reached a body weight of 5 to 7 kg or were between 26 and 30 days of age.

### Statistical analysis

Variance and covariance components were estimated using restricted maximum likelihood (REML) procedures, employing the Average Information Restricted Maximum Likelihood (AI-REML) algorithm implemented in the ASREML program [13]. The preliminary statistical analysis of NBA, LTBW, NPW, and LTWW utilized a single trait mixed animal repeatability model. The mixed animal repeatability model for each trait included the fixed effects of contemporary groups (first farrowing year-seasons), additive genetic group of the sow (based on the Y fraction of the sow), sow heterosis as a function of the heterozygosity of the sow (probability of alleles of different breeds at a single locus of the sow), litter heterosis as a function of the heterozygosity of the litter (probability of alleles of different breeds at a single locus of the litter), as well as covariates for age at first farrowing (ranging from 12 to 18 months) and days to weaning (applicable to NPW and LTWW only). The random effects in the single trait mixed repeatability model were sow, boar, and residual. The single trait mixed repeatability model in matrix notation is as follows:


$$y=\mathrm{Xb}+Z_{\mathrm{ga}}g_{a}+Z_{\mathrm{gn}}g_{n}+Z_{a}a_{a}+Wp_{e}+e$$

where **y** is a vector of records for NBA, LTBW, NPW, or LTWW, **b** is a vector of first farrowing year-seasons, covariates for age at first farrowing (mo), parity of sow, and days to weaning (d), **g_a_** is a vector of regression additive genetic group effects (difference between Y and L as a function of Y fraction), **g_n_** is a vector of heterosis effects of the sow and the litter, **a_a_** is a vector of random animal additive genetic effects, **X** is an incidence matrix of ones and zeroes that relates sow records to elements of vector **b**, **p_e_** is a vector of random permanent environment effects uncorrelated to animal additive genetic effects, **Z_ga_** is an incidence matrix of expected Y fractions of sows that relates sow records to elements of vector **g_a_**, **Z_gn_** is an incidence matrix of heterozygosities of the sow and the litter that relates sow records to elements of vector **g_n_**, **Z_a_** is an incidence matrix of ones and zeroes that relates sow records to elements of vector **a_a_**, **W** is an incidence matrix of ones and zeroes that relates sow records to elements of vector **p_e_**, and **e** is a vector of residuals. Expectations and (co)variance matrices of random variables in the mixed repeatability model were:


$$\begin{array}{l} E\;[y]=X_{b}+Zg_{a}+Z_{\mathrm{gn}}\;g_{n}\\ V\left[\begin{array}{c} a_{a}\\ p_{e}\\ e \end{array}\right]=\left[\begin{array}{ccc} A\sigma_{a}^{2} & 0 & 0\\ 0 & I\sigma_{\mathrm{pe}}^{2} & 0\\ 0 & 0 & I\sigma_{e}^{2} \end{array}\right] \end{array}$$

where **A** is the is the additive relationship matrix among all animals in the pedigree file (19,824 animals, 1,829 sires, and 4,473 dams), **I** represents identity matrices for permanent environmental and residual effects, $\sigma_{a}^{2}$ is the additive genetic variance, $\sigma_{\mathrm{pe}}^{2}$ is the permanent environmental variance, and $\sigma_{e}^{2}$ is the residual variance. Covariances between random effects were assumed to be zero.

Multiple trait analyses were conducted for two data sets. The first dataset included NBA, LTBW, NPW, and LTWW records from the first to the fourth parity. The NBA, LTBW, NPW, and LTWW records from different parities were considered to be different traits. Thus, a 4-trait mixed animal model analysis was conducted for each reproduction trait (NBA, LTBW, NPW, and LTWW). The 4-trait mixed animal model in matrix notation was as follows:


$$y=\mathrm{Xb}+Z_{\mathrm{ga}}g_{a}+Z_{\mathrm{gn}}g_{n}+Z_{a}a_{a}+e$$

where **y** is the vector of records for each reproduction trait (NBA, LTBW, NPW, or LTWW) in parities 1 to 4, **b** is a vector of contemporary group (first farrowing year-season) effects and covariates for age at first farrowing (mo) and days to weaning (d), **g_a_** is a vector of regression additive genetic group effects (difference between Y and L as a function of Y fraction), **g_n_** is a vector of heterosis effects of the sow and the litter, **a_a_** is a vector of random animal additive genetic effects, **e** is a vector of random residuals, **X** is an incidence matrix of ones and zeroes that relates sow records to elements of vector **b**, **Z_ga_** is an incidence matrix of expected Y fractions of sows that relates sow records to elements of vector **g_a_**, **Z_gn_** is an incidence matrix of heterozygosities of the sow and the litter that relates sow records to elements of vector **g_n_**, and **Z_a_** is an incidence matrix of ones and zeroes that relates sow records to elements of vector **a_a_**. The assumptions of the 4-trait mixed animal model were as follows:


$$\begin{array}{l} E\;[y]=\mathrm{Xb}+Z_{\mathrm{ga}}g_{a}+Z_{\mathrm{gn}}g_{n}\\ V\left[\begin{array}{c} a_{a}\\ e \end{array}\right]=\left[\begin{array}{cc} G_{a} & 0\\ 0 & R \end{array}\right] \end{array}$$

where **G_a_** = **G_0_** ⊗ **A**, **G_0_** is a 4×4 matrix of additive genetic variances and covariances for a single reproduction trait (NBA, LTBW, NPW, or LTWW) in parities 1 to 4, ⊗ is direct product, and A is the additive relationship matrix among all animals in the pedigree file (19,824 animals, 1,829 sires, and 4,473 dams). The pedigree file included all sows, all boars mated to L sows (L boars for purebred matings; Y boars for F1 LY crossbred matings), Y sows (Y boars for purebred matings; L boars for F1 YL crossbred matings), and LY and YL sows (mated only to D boars to produce three-breed terminal crossbred pigs for market), and all known relatives. Lastly, matrix **R** = **R_0_** ⊗ **I**, where **R_0_** is a 4×4 matrix of residual variances and covariances for a single reproduction trait (NBA, LTBW, NPW, or LTWW) in parities 1 to 4, and **I** is an identity matrix.

The estimation of heritabilities, additive genetic correlations, and phenotypic correlations involves analyzing the variance and covariance components associated with the four parities for all reproduction traits. Heritability (h^2^) is a measure of the proportion of the phenotypic variance attributed to additive genetic factors, calculated as the ratio of the additive genetic variance ( $\sigma_{\text{a}}^{2}$) to the phenotypic variance ( $\sigma_{\text{a}}^{2}+\sigma_{\text{e}}^{2}$). The additive genetic variance ( $\sigma_{\text{a}}^{2}$) represents the variability arising from additive genetic differences among individuals, while the environmental variance ( $\sigma_{\text{e}}^{2}$) captures the variability due to non-genetic factors. To assess the additive genetic relationship between traits x and y, the additive genetic covariance (cov_a(x,y)_), which quantifies the joint variability due to additive genetic factors, is divided by the square root of the product of the additive genetic variances (var_a(x)_ and var_a(y)_) for each respective trait. This calculation yields the additive genetic correlation (r_a_) between the two traits. Similarly, this methodology can be applied to determine phenotypic correlations, which capture the overall association between traits, including both genetic and environmental influences.

Reproduction records (NBA, LTBW, NPW, and LTWW) were classified into three groups in the second dataset. Group 1 included first parity records only, group 2 contained second parity records only, and group 3 contained sums of NBA, LTBW, NPW, and LTWW records from the third to the last parity. Reproduction records in parities 1, 2, and 3+ (third and later parities) were considered to be different traits. Thus, a 3-trait mixed animal model was utilized to analyze each reproduction trait in the second dataset. The expression for the 3-trait mixed animal model is as in equation 2. The assumptions for the 3-trait mixed animal model were as follows:


$$\begin{array}{l} E\;[y]=\mathrm{Xb}+Z_{\mathrm{ga}}g_{a}+Z_{\mathrm{gn}}g_{n}\\ V\left[\begin{array}{c} a_{a}\\ e \end{array}\right]=\left[\begin{array}{cc} G_{a} & 0\\ 0 & R \end{array}\right] \end{array}$$

where **G_a_** = **G_0_** ⊗ **A**, **G_0_** is a 3×3 matrix of additive genetic variances and covariances for a single reproduction trait (NBA, LTBW, NPW, or LTWW) in parities 1 to 3+, matrix **R** = **R_0_** ⊗ **I**, **R_0_** is a 3×3 matrix of residual variances and covariances for a single reproduction trait (NBA, LTBW, NPW, or LTWW) in parities 1 to 3+, and all other vectors and matrices are as defined for the 4-trait mixed animal model used for the first dataset. Similarly, the estimated variance and covariance components were used to compute heritabilities, additive genetic correlations, and phenotypic correlations between the three parities for the four reproduction traits.

## RESULTS

Table 1 presents numbers of observations, means, and standard deviations for NBA, LTBW, NPW, and LTWW across different parities in the swine population. This information provides a comprehensive overview of the actual reproductive performance of sows for the four reproductive traits as parity number increased. The means and standard deviations for parity 1 were 9.43±2.65 piglets for NBA, 14.56±4.44 kg for LTBW, 8.54±2.36 piglets for NPW, and 59.68±19.83 kg for LTWW. The means of the four reproductive traits increased in the second parity (10.05±2.62 piglets for NBA, 16.52±4.64 kg for LTBW, 9.48±2.14 piglets for NPW, and 70.59±20.79 kg for LTWW). These means further increased in parity 3 (10.64±2.52 piglets for NBA, 17.59±4.86 kg for LTBW, 9.51±1.95 piglets for NPW, and 74.08±20.92 kg for LTWW), but the means in parity 4 were similar to those in parity 3 (10.70±2.52 piglets for NBA, 17.65±4.86 kg for LTBW, 9.67±2.01 piglets for NPW, and 74.54±20.13 kg for LTWW). Lastly, the values for parity 3+ representing sums of NBA, LTBW, NPW, and LTWW records from the third to the last parity were 43.57±22.60 piglets for NBA, 69.76± 36.64 kg for LTBW, 39.10±20.24 piglets for NPW, and 293.8± 164.20 kg for LTWW.

The estimates of additive genetic, permanent environmental, temporary environmental, and phenotypic variance components for NBA, LTBW, NPW, and LTWW in parities 1 to 4 computed using a single trait mixed animal repeatability model are presented in Table 2. This analysis revealed that the largest portion of the variability for NBA was attributed to temporary environmental effects (90%), followed by the permanent environmental effect (8%), and the additive genetic effect (2%). Similarly, variance estimates for temporary environmental effects for LTBW, NPW, and LTWW were higher (85% for NBA, 93% for NPW, and 85% for LTWW) than variance estimates for permanent environmental effects (5% for LTBW, 4% for NPW, and 3% for LTWW) and additive genetic effects (10% for LTBW, 4% for NPW, and 11% for LTWW). Consequently, heritability estimates for the four reproduction traits in the first four parities were low, ranging from 0.02±0.01 for NBA to 0.11±0.01 for LTWW (Table 2). These estimates of heritability revealed a predominant influence of temporary environmental effects on the variability of reproduction traits, emphasizing the importance of environmental factors in the expression of these traits.

The estimates of additive genetic, environmental, and phenotypic variance components for NBA, LTBW, NPW, and LTWW in parities 1 to 4 computed using a 4-trait mixed animal model are presented in Table 3. Additive genetic variances ranged from 0.20±0.08 piglets^2^ in parity 2 to 0.51±0.13 piglets^2^ in parity 4 for NBA, from 0.98±0.26 kg^2^ in parity 2 to 3.10±0.43 kg^2^ in parity 3 for LTBW, from 0.17±0.07 piglets^2^ in parity 4 to 0.42±0.08 piglets^2^ in parity 1 for NPW, and from 56.85±6.37 kg^2^ in parity 1 to 73.82±7.67 kg^2^ in parity 2 for LTWW. Environmental variances ranged from 5.38±0.12 piglets^2^ in parity 2 to 6.34±0.12 piglets^2^ in parity 1 for NBA, from 13.77±0.32 kg^2^ in parity 2 to 17.46±0.50 kg^2^ in parity 4 for LTBW, from 3.53±0.08 piglets^2^ in parity 3 to 5.01±0.10 piglets^2^ in parity 1 for NPW, and from 281.23±8.21 kg^2^ in parity 4 to 298.11±6.65 kg^2^ in parity 1 for LTWW. Phenotypic variances ranged from 5.58±0.09 piglets^2^ in parity 2 to 6.64±0.10 piglets^2^ in parity 1 for NBA, from 14.74±0.25 kg^2^ in parity 2 to 19.95±0.38 kg^2^ in parity 4 for LTBW, from 3.74±0.07 piglets^2^ in parity 3 to 5.43±0.08 piglets^2^ in parity 1 for NPW, and from 339.00±6.46 kg^2^ in parity 4 to 364.60± 6.06 kg^2^ in parity 1 for LTWW. These estimates of variance components show the variability in additive genetic, environmental, and phenotypic variances across parities, highlighting the importance of considering parity-specific estimates when analyzing reproduction traits in swine populations.

Table 4 shows estimates of heritabilities, additive genetic correlations, and phenotypic correlations between NBA, LTBW, NPW, and LTWW in the first 4 parities of sows. Estimates were computed using dataset 1 and a 4-trait mixed animal model. Heritability estimates tended to increase as the number of parities increased for NBA (from 0.05±0.01 in parity 1 to 0.09±0.02 in parity 4) and LTBW (from 0.10± 0.02 in parity 1 to 0.13±0.02 in parity 4), they tended to decrease for NPW (from 0.08±0.01 in parity 1 to 0.04±0.02 in parity 4), but showed little change for LTWW (from 0.16±0.02 in parity 1 to 0.17±0.02 in parity 4). Overall, heritability estimates were low for NBA and NPW and close to moderate for LTBW and LTWW. Barring the heritability estimate for LTBW in the first parity, estimates of heritabilities for numbers of piglets at birth and at weaning were consistently lower than heritabilities for litter weights at birth and at weaning in all parities. Heritability estimates varied across traits and parities, tending to increase for NBA and LTBW and to decrease for NPW as parity number increased, whereas they tended to remain relatively stable for LTWW. Heritability estimates for litter weights were consistently higher than those for litter sizes, indicating a stronger genetic influence on litter weights.

Estimates of additive genetic correlations between NBA, LTBW, NPW, and LTWW in the first four parities were positive and moderate to high for all reproduction traits. Values ranged from 0.29±0.24 between parities 1 and 2 to 0.96±0.20 between parities 2 and 4 for NBA, 0.72±0.01 between parities 1 and 4 to 0.93±0.07 between parities 3 and 4 for LTBW, 0.52 ±0.20 between parities 1 and 4 to 0.91±0.17 between parities 2 and 3 for NPW, and 0.68±0.08 between parities 1 and 3 to 0.99±0.05 between parities 3 and 4 for LTWW. Conversely, phenotypic correlations between the parities 1 to 4 were positive and low for the four reproduction traits, with values ranging from 0.05±0.01 between parities 1 and 2, 1 and 3, and 2 and 3 for NPW to 0.27±0.01 between parities 3 and 4 for LTWW. Additive genetic correlations between reproduction traits were positive and moderate to high across parities, indicating a shared genetic influence. In contrast, phenotypic correlations between parities were positive but low, suggesting the presence of additional environmental or management factors influencing the observed correlations.

Table 5 presents estimates of heritabilities, additive genetic correlations, and phenotypic correlations between NBA, LTBW, NPW, and LTWW in parities 1, 2, and 3+ using dataset 2 and a 3-trait mixed animal model. As indicated above, parity 3+ contains sums of NBA, LTBW, NPW, and LTWW records from the third to the last parity of a sow. Heritability estimates were low for NBA, LTBW, NPW, and LTWW in all three parities, except for the moderate heritability estimate for LTWW in parity 3+ (0.23±0.03). The remaining heritability estimates ranged from 0.02±0.01 for NPW in parity 1 to 0.10 ±0.02 for NPW in parity 3+ and LTWW in parity 2. Additive genetic correlations between parities were positive and low for NBA (0.11±0.19 between parities 1 and 3+ to 0.51±0.18 between parities 2 and 3+), positive and low to moderate for LTBW (0.12±0.17 between parities 1 and 3+ to 0.83±0.11 between parities 1 and 2), and positive and moderate to high for NPW (0.66±0.33 between parities 1 and 2 to 0.96±0.17 between parities 2 and 3+) and LTWW (0.80±0.08 between parities 1 and 3+ to 0.99±0.07 between parities 2 and 3+). Conversely, phenotypic correlations between parities 1, 2, and 3+ were positive and low for all four reproductive traits. Values ranged from 0.05±0.01 between parities 1 and 3+ for NBA to 0.18±0.01 between parities 2 and 3+ for LTWW. Heritability estimates were low for reproductive traits across parities, except for a moderate heritability value obtained for LTWW in parity 3+. Additive genetic correlations exhibited various patterns, with low to moderate correlations between parities for NBA and LTBW, and moderate to high correlations for NPW and LTWW. Phenotypic correlations were consistently positive but low across parities for all four reproductive traits.

## DISCUSSION

The estimates of additive genetic, environmental, and phenotypic variance components for NBA, LTBW, NPW, and LTWW obtained from dataset 1 using the 4-trait mixed animal model varied across parities. However, there was no consistent pattern regarding which parity exhibited the minimum and maximum values. These fluctuations in variance component estimates among parities present an intriguing challenge when attempting to interpret their broader implications. This challenge stems from the fact that these estimates are influenced by the unique genetic and environmental characteristics inherent to each specific swine population. Understanding the underlying factors driving the observed differences in variance component estimates across parities is crucial for accurate genetic evaluations and informed breeding decisions. The genetic architecture, including the presence of specific genes or gene combinations, may contribute to the variations observed. Additionally, environmental factors such as management practices, nutrition, and housing conditions can significantly impact the expression of reproductive traits. Consequently, the interplay between genetics and the environment complicates the interpretation of variance component estimates.

Further investigation is necessary to unravel the specific mechanisms influencing differences in variance components among parities. This may involve examining additional factors, such as maternal effects, gene-by-environment interactions, and potential genotype-environment correlations. By delving deeper into the underlying genetic and environmental influences, we can enhance our understanding of the complex dynamics governing reproductive traits in swine populations. Therefore, caution should be exercised when making broad generalizations based solely on the observed differences in variance component estimates among parities. Instead, a comprehensive analysis considering the unique characteristics of each swine population is warranted to unravel the underlying factors and provide a more nuanced interpretation of the results.

The heritability estimates for NBA, LTBW, NPW, and LTWW in our study were compared to previous research conducted in different populations, revealing both similarities and differences [14–17]. The heritability estimates from our study were slightly higher than those reported in a previous study involving a Landrace-Large White multibreed population in Northern Thailand [3]. However, they were comparatively lower than estimates reported for Large White populations in the United States and Mexico [18], as well as for Duroc, Landrace, and Yorkshire populations in South Korea [9], Iberian sows in Spain [7], and Black Slavonian pigs in Slovenia [8]. This variation in heritability estimates across studies highlights the influence of genetic and environmental factors specific to each population. Interestingly, our results revealed an increasing trend in heritability estimates as parity numbers increased for NBA, LTBW, and LTWW. This finding suggests that these traits may exhibit higher rates of genetic change in later parities. This trend agrees with similar observations reported in other studies, such as those conducted on Czech Landrace and Slovak Meaty breed in the Czech Republic and the Slovak Republic [19], Dutch Landrace in The Netherlands [20], Landrace pigs in Spain [21], German Landrace and Pietrain swine in Germany [22], and Landrace and Large White pigs in Japan [23].

Heritability estimates for NBA in different parities differed from those obtained by Skorput et al [8] in Black Slavonian pigs. Our estimates for NBA in parity 1 (0.04±0.01) and parity 3+ (0.09±0.02) were higher than those reported by Skorput et al [8], while the estimate for NBA in parity 2 (0.04±0.01) was lower. Furthermore, our heritability estimates for NBA in parity 1 were lower than estimates reported for Landrace swine populations in Germany [22,24], Spain [21], the Netherlands [20], Iberian swine populations in Spain [7], and commercial swine populations in South Korea [9]. Notably, most heritability estimates obtained using the 3-trait and 4-trait mixed animal models were higher than those obtained with the repeatability model. This indicates that the use of more comprehensive models accounting for additional sources of variation provides more accurate and reliable estimates of heritability. Although the heritability estimates for NPW were lower in parities 3 and 4 compared to parities 1 and 2 in dataset 1, there was a steady increase from parity 1 to 3+ in dataset 2. These results suggest that selection for NPW may exhibit different patterns of genetic change across parities.

Our findings demonstrate the complex nature of heritability estimates for NBA, LTBW, NPW, and LTWW, which are influenced by genetic and environmental factors specific to each population. The observed trend of increasing heritability estimates as parity number increases for certain traits highlights the potential for genetic improvement in later parities. However, caution must be exercised when making comparisons across studies due to differences in population characteristics and modeling approaches. Collectively, these findings contribute to our understanding of the genetic basis of reproductive traits in swine populations and provide insights for future breeding and selection strategies.

The analysis of additive genetic correlations between NBA, LTBW, NPW, and LTWW across parities from datasets 1 and 2 revealed intriguing patterns and provided insights into the reproductive traits of the swine population under study. Notably, we found a higher level of additive genetic association between reproduction traits in the first parity and subsequent parities at weaning than between reproduction traits at birth. This observation suggests that the genetic influences on these traits become more evident as sows progress through subsequent parities. In contrast, phenotypic correlations between reproduction traits in the first and subsequent parities were consistently low at both birth and weaning, indicating limited phenotypic associations between these traits in this particular swine population. While these phenotypic correlations provide less information, additive genetic correlations offer valuable insights into the genetic associations among the traits.

Comparing our estimates to those from previous studies, we found that the additive genetic correlation between NBA in the first parity and the sum from the third to the last parity in this population (0.11±0.19) was lower than those reported (0.84±0.05) for Iberian pigs in Spain [7] and a Landrace-Yorkshire multibreed swine population (0.19±0.09) in Northern Thailand [25]. However, the estimates for additive genetic correlations between NBA across parities were consistent with values obtained from diverse populations, including Swedish Landrace, Large White, their reciprocal crossbreds, German Landrace, and a backcross of Large White and German Landrace with Large White in Croatia [12]. Similarly, the additive genetic correlations between NBA across parities in our study aligned with estimates computed for Landrace in The Netherlands [20] (0.55 to 0.99) and Spain [21] (0.49±0.07 to 0.92±0.04), Landrace and Yorkshire in Australia [11] (0.61±0.30 to 0.62±0.19), Large White in the USA and Mexico [18] (0.74 to 0.95), and Black Slavonian pigs in Croatia [8] (0.05 to 0.96).

The consistency of these estimates across multiple swine populations and different parities suggests that additive genetic correlations across parities tend to have moderate to high positive values. This implies that the information from earlier parities can greatly enhance the accuracy of sow additive genetic predictions in later parities. Therefore, selecting replacement sows based on reproductive information from all parities is expected to improve the lifetime productivity of sows in terms of the number of piglets and litter weights. These findings provide valuable insights for breeding programs and selection strategies aimed at enhancing sow lifetime productivity. By considering the additive genetic associations across parities, breeders can make more informed decisions and improve the efficiency of genetic selection. Furthermore, the observed patterns of genetic correlations shed light on the underlying genetic architecture of reproductive traits in this swine population and contributed to our understanding of the complex interplay between genetics and reproduction in pigs.

Our investigation into the heritability values of NBA, LTBW, NPW, and LTWW across different parities using MTM has revealed important insights into the genetic improvement potential of these reproductive traits in this Thai swine population. Importantly, we observed that the heritability estimates obtained with the MTMs were consistently higher than those obtained with the repeatability model for all four traits. This indicates that utilization of a MTM has a greater impact on genetic progress for NBA, LTBW, NPW, and LTWW than the repeatability model in this swine population. The higher heritability values, along with the positive moderate to high additive genetic correlations observed between parities for these traits, further emphasize the need to consider first, second, and later parities as distinct traits in genetic evaluations. This finding is consistent with previous research conducted by Roehe and Kennedy [10], Hermesch et al [11], Noguera et al [21], Serenius et al [5], Oh et al [9], Ye et al [15], Lopez et al [16], Ogawa et al [23], and Konta et al [26] stressing the importance of treating reproductive records from different parities as separate traits. This distinction arises from the fact that different sets of genes govern the hormonal and physiological conditions of first parity and older sows [7,11, 20,21,27]. To effectively improve NBA, LTBW, NPW, and LTWW in this Thai swine population, it is recommended to adopt a multiple trait genetic evaluation approach that treats each parity as a distinct trait. Such an approach is more suitable for accurately capturing the genetic variability and potential for improvement of these reproductive traits throughout the lifetime of the sows.

In conclusion, this study demonstrates that utilizing a multiple trait model leads to higher estimates of heritabilities for NBA, LTBW, NPW, and LTWW in this swine population compared to a repeatability model. This underscores the significance of considering first, second, and later parities as separate traits within swine genetic improvement programs. The positive additive genetic correlations observed among different parities for all reproduction traits provide a basis for optimizing breeding strategies and enhancing the lifetime productivity of sows. By adopting a multiple trait approach and acknowledging the distinct genetic contributions to each parity, swine breeders in Thailand can make more accurate selection decisions while achieving sustainable improvements in reproductive performance. Further research involving multiple swine operations would contribute to the continued development of effective and sustainable swine breeding programs in the country, thus further advancing the field of swine genetic evaluation and selection.

## Figures and Tables

**Table 1:** Summary statistics for NBA, LTBW, NPW, and LTWW across different parities

Parity	NBA (piglets)	LTBW (kg)	NPW (piglets)	LTWW (kg)
1	9.43±2.65^1)^ (9,732)^2)^	14.56±4.44 (9,611)	8.54±2.36 (9,826)	59.68±19.83 (9,828)
2	10.05±2.62 (8,255)	16.52±4.64 (8,044)	9.48±2.14 (8,002)	70.59±20.79 (7,859)
3	10.64±2.52 (7,094)	17.59±4.86 (6,960)	9.51±1.95 (6,584)	74.08±20.92 (6,782)
4	10.70±2.52 (6,107)	17.65±4.86 (5,963)	9.67±2.01 (5,999)	74.54±20.13 (5,783)
3+ ^3)^	43.57±22.60 (7,240)	69.76±36.64 (7,216)	39.10±20.24 (7,069)	293.8±164.20 (7,071)

NBA, number of piglets born alive; LTBW, litter birth weight; NPW, number of piglets weaned; LTWW, litter weaning weight.

1) Mean±standard deviation.

2) Number of records.

3) Sum from the third to the last parity.

**Table 2:** Estimates of additive genetic, permanent environmental, temporary environmental, and phenotypic variances as well as heritabilities for NBA, LTBW, NPW, and LTWW using a single trait mixed animal repeatability model

Trait	Variance component^1)^				h^2^

	$\sigma_{a}^{2}$	$\sigma_{pe}^{2}$	$\sigma_{e}^{2}$	$\sigma_{p}^{2}$	
NBA (piglets^2^)	0.08±0.02	0.29±0.03	3.42±0.04	3.80±0.04	0.02±0.01
LTBW (kg^2^)	2.11±0.16	1.07±0.13	17.35±0.12	20.53±0.15	0.10±0.01
NPW (piglets^2^)	0.17±0.02	0.16±0.02	4.23±0.03	4.56±0.03	0.04±0.01
LTWW (kg^2^)	41.16±2.93	10.85±2.30	306.67±2.21	358.70±2.61	0.11±0.01

NBA, number of piglets born alive; LTBW, litter birth weight; NPW, number of piglets weaned; LTWW, litter weaning weight.

1) $\sigma_{a}^{2}$, additive genetic variance; $\sigma_{pe}^{2}$, permanent environmental variance; $\sigma_{e}^{2}$, temporary environmental variance; $\sigma_{p}^{2}$, phenotypic variance; h^2^, heritability.

**Table 3:** Estimates of additive genetic, environmental, and phenotypic variances for NBA, LTBW, NPW, and LTWW in parities 1 to 4 using a 4-trait mixed animal model

Trait	Additive genetic variance	Environmental variance	Phenotypic variance
Number of piglets born alive (piglets^2^)			
NBA1	0.30±0.09	6.34±0.12	6.64±0.10
NBA2	0.20±0.08	5.38±0.12	5.58±0.09
NBA3	0.42±0.10	5.70±0.13	6.13±0.10
NBA4	0.51±0.13	5.42±0.16	5.93±0.12
Litter birth weight (kg^2^)			
LTBW1	1.64±0.28	15.44±0.32	17.07±0.25
LTBW2	0.98±0.26	13.77±0.32	14.74±0.25
LTBW3	3.10±0.43	16.15±0.44	19.26±0.34
LTBW4	2.49±0.46	17.46±0.50	19.95±0.38
Number of piglets weaned (piglets^2^)			
NPW1	0.42±0.08	5.01±0.10	5.43±0.08
NPW2	0.38±0.08	4.11±0.09	4.49±0.07
NPW3	0.20±0.06	3.53±0.08	3.74±0.07
NPW4	0.17±0.07	3.81±0.09	3.98±0.07
Litter weaning weight (kg^2^)			
LTWW1	56.85±6.37	298.11±6.65	355.00±5.23
LTWW2	73.82±7.67	290.81±7.56	364.60±6.06
LTWW3	62.46±9.41	286.21±9.65	348.70±7.20
LTWW4	57.82±7.85	281.23±8.21	339.00±6.46

NBA1, number of piglets born alive in the first parity; NBA2, number of piglets born alive in the second parity; NBA3, number of piglets born alive in the third parity; NBA2, number of piglets born alive in the fourth parity; LTBW1, litter birth weight in the first parity; LTBW2, litter birth weight in the second parity; LTBW3, litter birth weight in the fourth parity; LTBW4, litter birth weight in the fourth parity; NPW1, number of piglets weaned in the first parity; NPW2, number of piglets weaned in the second parity; NPW3, number of piglets weaned in the third parity; NPW4, number of piglets weaned in the fourth parity; LTWW1, litter weaning weight in the first parity; LTWW2, litter weaning weight in the second parity; LTWW3, litter weaning weight in the third parity; LTWW4, litter weaning weight in the fourth parity.

**Table 4:** Heritabilities (±SE; diagonal), additive genetic correlations (±SE; above diagonal), and phenotypic correlations (±SE; below diagonal) for NBA and LTBW in parities 1 to 4 using a 4-trait mixed animal model

Trait	Parity			

	1	2	3	4
Number of piglets born alive	NBA1	NBA2	NBA3	NBA4
NBA1	0.05±0.01	0.29±0.24	0.70±0.16	0.50±0.18
NBA2	0.09±0.01	0.04±0.01	0.86±0.2	0.96±0.20
NBA3	0.10±0.01	0.09±0.01	0.07±0.02	0.94±0.13
NBA4	0.12±0.01	0.14±0.01	0.15±0.01	0.09±0.02
Litter birth weight	LTBW1	LTBW2	LTBW3	LTBW4
LTBW1	0.10±0.02	0.81±0.12	0.92±0.07	0.72±0.1
LTBW2	0.16±0.01	0.07±0.02	0.93±0.10	0.84±0.12
LTBW3	0.17±0.01	0.15±0.01	0.16±0.02	0.93±0.07
LTBW4	0.13±0.01	0.15±0.01	0.21±0.01	0.13±0.02
Number of piglets weaned	NPW1	NPW2	NPW3	NPW4
NPW1	0.08±0.01	0.62±0.13	0.88±0.16	0.52±0.20
NPW2	0.05±0.01	0.08±0.02	0.91±0.17	0.82±0.19
NPW3	0.05±0.01	0.05±0.01	0.05±0.02	0.75±0.23
NPW4	0.07±0.01	0.09±0.01	0.10±0.01	0.04±0.02
Litter weaning weight	LTWW1	LTWW2	LTWW3	LTWW4
LTWW1	0.16±0.02	0.82±0.06	0.68±0.08	0.74±0.08
LTWW2	0.16±0.01	0.20±0.02	0.87±0.07	0.92±0.06
LTWW3	0.13±0.01	0.14±0.01	0.18±0.03	0.99±0.05
LTWW4	0.13±0.01	0.17±0.01	0.27±0.01	0.17±0.02

SE, standard error; NBA1, number of piglets born alive in the first parity; NBA2, number of piglets born alive in the second parity; NBA3, number of piglets born alive in the third parity; NBA2, number of piglets born alive in the fourth parity; LTBW1, litter birth weight in the first parity; LTBW2, litter birth weight in the second parity; LTBW3, litter birth weight in the fourth parity; LTBW4, litter birth weight in the fourth parity; NPW1, number of piglets weaned in the first parity; NPW2, number of piglets weaned in the second parity; NPW3, number of piglets weaned in the third parity; NPW4, number of piglets weaned in the fourth parity; LTWW1, litter weaning weight in the first parity; LTWW2, litter weaning weight in the second parity; LTWW3, litter weaning weight in the third parity; LTWW4, litter weaning weight in the fourth parity.

**Table 5:** Heritabilities (±SE; diagonal), additive genetic correlations (±SE; above diagonal), and phenotypic correlations (±SE; below diagonal) for NBA, LTBW, NPW, and LTWW in parities 1, 2, and 3+ (third and later parities) using a 3-trait mixed animal model

Trait	Parity		

	1	2	3+
Number of piglets born alive	NBA1	NBA2	NBA3+
NBA1	0.04±0.01	0.54±0.21	0.11±0.19
NBA2	0.11±0.01	0.04±0.01	0.51±0.18
NBA3+	0.05±0.01	0.07±0.01	0.09±0.02
Litter birth weight	LTBW1	LTBW2	LTBW3+
LTBW1	0.08±0.02	0.83±0.11	0.12±0.17
LTBW2	0.17±0.01	0.07±0.02	0.23±0.18
LTBW3+	0.08±0.01	0.08±0.01	0.06±0.02
Number of piglets weaned	NPW1	NPW2	NPW3+
NPW1	0.02±0.01	0.66±0.33	0.83±0.26
NPW2	0.04±0.01	0.04±0.01	0.96±0.17
NPW3+	0.08±0.01	0.10±0.01	0.10±0.02
Litter weaning weight	LTWW1	LTWW2	LTWW3+
LTWW1	0.08±0.01	0.87±0.09	0.80±0.08
LTWW2	0.13±0.01	0.10±0.02	0.99±0.07
LTWW3+	0.13±0.01	0.18±0.01	0.23±0.03

SE, standard error; NBA1, number of piglets born alive in the first parity; NBA2, number of piglets born alive in the second parity; NBA3+, sum of number of piglets born alive from the third to the last parity; LTBW1, litter birth weight in the first parity; LTBW2, litter birth weight in the second parity; LTBW3+, sum of litter birth weights from the third to the last parity; NPW1, number of piglets weaned in the first parity; NPW2, number of piglets weaned in the second parity; NPW3+, sum of number of piglets weaned from the third to the last parity; LTWW1, litter weaning weight in the first parity; LTWW2, litter weaning weight in the second parity; LTWW3+, sum of litter weaning weights from the third to the last parity.

## Data Availability

The data used in this study will be shared upon a reasonable request to the corresponding author.
